# Laparoscopic appendicectomy for suspected mesh-induced appendicitis after laparoscopic transabdominal preperitoneal polypropylene mesh inguinal herniorraphy

**DOI:** 10.4103/0972-9941.62532

**Published:** 2010

**Authors:** Jason M Jennings, Philip CH Ng

**Affiliations:** Department of General Surgery, Lewisham Hospital NHS Trust, Lewisham High Street, London, SE13 6LH UK

**Keywords:** Appendicectomy, appendicitis, inguinal herniorraphy, laparoscopy, mesh, polypropylene

## Abstract

Laparoscopic inguinal herniorraphy via a transabdominal preperitoneal (TAPP) approach using Polypropylene Mesh (Mesh) and staples is an accepted technique. Mesh induces a localised inflammatory response that may extend to, and involve, adjacent abdominal and pelvic viscera such as the appendix. We present an interesting case of suspected Mesh-induced appendicitis treated successfully with laparoscopic appendicectomy, without Mesh removal, in an elderly gentleman who presented with symptoms and signs of acute appendicitis 18 months after laparoscopic inguinal hernia repair. Possible mechanisms for Mesh-induced appendicitis are briefly discussed.

## INTRODUCTION

We report a case of appendicitis intimately associated with a previous laparoscopic Mesh herniorraphy raising the suspicion that an implanted Mesh may cause appendicitis. A literature search using the terms ‘mesh’, ‘polypropylene mesh’, ‘appendicitis’, ‘appendicectomy’ and ‘infection’ was conducted, to determine if similar cases had been previously reported, and to determine whether Mesh infection is common after appendicitis or appendicectomy. No cases suggesting that Mesh induces appendicitis have been reported. In addition, the incidence of mesh infection following appendicitis appears to be extremely low, with only one case report describing Mesh infection following perforated appendicitis eight years after laparoscopic (TAPP) inguinal herniorraphy, necessitating Mesh removal.[1]

## CASE REPORT

A 69-year-old man underwent successful laparoscopic inguinal hernia (TAPP) repair for a right indirect inguinal hernia. Apart from complaining of mild chronic right iliac fossa discomfort, persisting for some time after the repair, the patient made an uneventful recovery from his primary procedure. He presented to the Emergency Department 18 months later with signs and symptoms suggestive of acute appendicitis. He was prepared for the theatre and consented for diagnostic laparoscopy and appendicectomy. Following administration of antibiotics, an emergency diagnostic laparoscopy was performed and digitally recorded utilising the Karl Stortz AIDA II Video-endoscope System.

**Figure 1:**
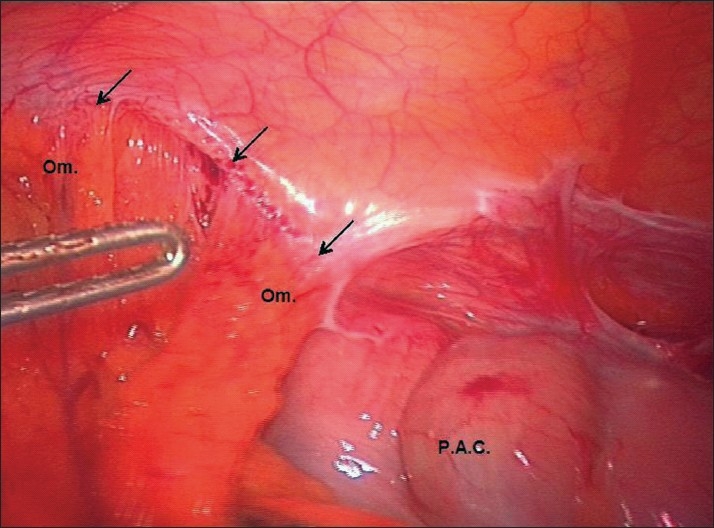
The undisturbed view of the structures visualised in the right iliac fossa at diagnostic laparoscopy. The greater omentum overlies the appendix and peritonealised Mesh and is clearly seen to be attached to the anterior abdominal wall. The black arrows identify the omental adhesions to the anterior abdominal wall that follow a line corresponding to the peritoneal closure from the TAPP repair. P.A.C. = Proximal Ascending Colon; Om. = Omentum

**Figure 2:**
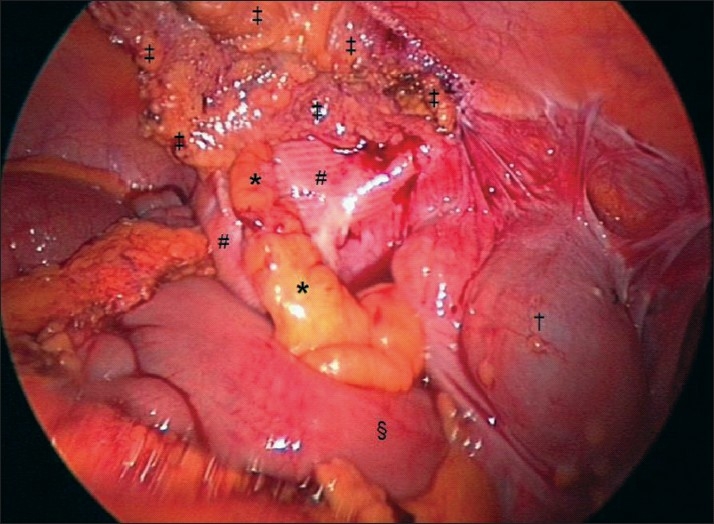
View of turgid appendix, overlying, and attached to, the peritonealised Mesh revealed after division of the covering omentum using bipolar diathermy. The omentum was adherent superiorly along a line corresponding to the line of peritoneal overlap and/or imperfectly covered areas of Mesh that may result after TAPP repair. * = Appendix adherent to peritonealised Mesh; # = peritonealised Mesh; ‡ = Remnant of omentum left attached to the anterior abdominal wall; † = Caecum; § = Terminal ileum

**Figure 3:**
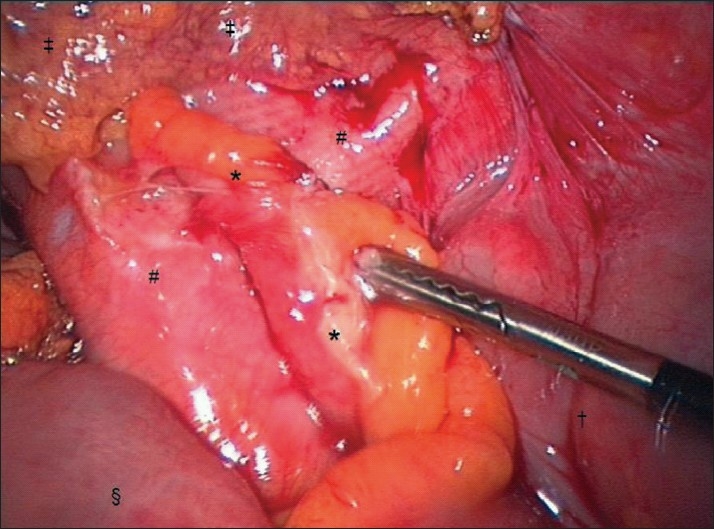
Close up image acquired with the video-endoscope zoom facility illustrating adhesions between the turgid appendix and peritonealised Mesh. * = Appendix adherent to peritonealised Mesh; # = peritonealised Mesh; ‡ = Remnant of omentum left attached to the anterior abdominal wall; † = Caecum; § = Terminal ileum

During diagnostic laparoscopy the greater omentum was found to be attached to the anterior abdominal wall, along a line corresponding to the peritoneal closure from the TAPP repair [Figure 1]. The omentum was carefully divided with bipolar diathermy taking care not to expose the underlying mesh. The omental adhesiolysis in the right iliac fossa revealed a small amount of free fluid and a turgid appendix overlying and adherent to the peritonealised Mesh from the previous right laparoscopic inguinal herniorraphy [Figures 2 and 3]. The turgid appendix was carefully dissected off the peritonealised Mesh using sharp and blunt dissection; again taking care not to expose the underlying Mesh. An uncomplicated laparoscopic appendicectomy was performed, and a local washout with normal saline completed the procedure. As no Mesh was found to be exposed during the procedure the decision was made to leave the Mesh *in situ* and a short course of antibiotics prescribed postoperatively. The patient made an uneventful recovery, and was discharged home the following day without subsequent complication. Histology revealed severe acute suppurative appendicitis with early microscopic mucosal ulceration and perforation.

## DISCUSSION

Laparoscopic inguinal herniorraphy via the Transabdominal preperitoneal (TAPP) approach using polypropylene mesh (Mesh) and spiral staples is an acceptable technique that is used within our institution. Briefly, after establishing a pneumoperitoneum, the peritoneum is incised via a curved incision from the superior-medial to the lateral aspects of the inguinal region starting high and lateral to the lateral umbilical ligament. The pre-peritoneal space is opened by blunt and diathermy dissection to meticulously expose the Cooper's ligament, the epigastric vessels, hernia sac, spermatic cord (or round ligament), and the femoral canal, clearly identifying the region of the iliac vessels. The hernia sac is dissected off the cord, reduced or transected and a polypropylene mesh is cut to size and placed over the inguinal region, covering the defect. The Mesh is secured using the Protack^®^ spiral stapling device, and the detached peritoneum is then brought up, laterally to medially, and stapled in place, to completely cover the Mesh avoiding leaving defects that may expose the Mesh to the intra-abdominal viscera.

A fundamental mechanism responsible for affecting and strengthening the hernia repair is that Mesh induces a localised inflammatory response. This inflammatory response may subsequently result in fibrinous adhesions to adjacent organs such as the greater omentum, small and large bowel, the appendix, and the fallopian tubes and ovaries in females. While it is often recognised that serosal appendicitis may be induced by the inflammation of neighbouring tissues, for example, sigmoid diverticulitis, it is not known whether the inflammation of the appendix is induced by Mesh, either directly or as a result of local adhesions, leading to purulent appendicitis. It is conjectured that the inflammatory reaction resulting from the exposure of a small area of imperfectly covered Mesh, or the inflammation associated with healing that occurs along the line of the peritoneal overlap in a TAPP repair may induce acute or chronic serosal inflammation of the appendix that results in luminal occlusion, bacterial overgrowth and subsequent purulent appendicitis. Alternatively, appendiceal adhesions may result in a fixed conformation that may predispose to luminal obstruction, such as, at a site of kinking of the appendix.

Acute signs or symptoms of appendicitis, even after many months or years after laparoscopic herniorraphy should be taken seriously, and diagnostic laparoscopy should be considered early as a first-line investigation. Chronic right iliac fossa pain after laparoscopic (TAPP) inguinal herniorraphy, or indeed any other mesh repair, may be due to chronic serosal inflammation of the appendix that theoretically could progress to acute appendicitis.

However, caution is strongly advised when making such inferences, as appendicitis is common and the association may be a mere coincidence. Furthermore, studies aimed at determining whether such a causal relationship exists, may start with the hypothesis that appendicitis is found with greater frequency in patients who have had laparoscopic mesh repair of right inguinal hernias, compared to the general population or those patients who have had laparoscopic mesh repair of a left inguinal hernia.
